# Mind-blanking: when the mind goes away

**DOI:** 10.3389/fpsyg.2013.00650

**Published:** 2013-09-27

**Authors:** Adrian F. Ward, Daniel M. Wegner

**Affiliations:** Department of Psychology, Harvard UniversityCambridge, MA, USA

**Keywords:** consciousness, attention, perception, mind-wandering, mind-blanking, stimulus-independent thought, task-unrelated thought, reading comprehension

## Abstract

People often feel like their minds and their bodies are in different places. Far from an exotic experience, this phenomenon seems to be a ubiquitous facet of human life (e.g., Killingsworth and Gilbert, 2010). Many times, people's minds seem to go “somewhere else”—attention becomes disconnected from perception, and people's minds wander to times and places removed from the current environment (e.g., Schooler et al., 2004). At other times, however, people's minds may seem to go nowhere at all—they simply disappear. This mental state—mind-blanking—may represent an extreme decoupling of perception and attention, one in which attention fails to bring any stimuli into conscious awareness. In the present research, we outline the properties of mind-blanking, differentiating this mental state from other mental states in terms of phenomenological experience, behavioral outcomes, and underlying cognitive processes. Seven experiments suggest that when the mind seems to disappear, there are times when we have simply failed to monitor its whereabouts—and there are times when it is actually gone.

“Where is my mind?” (The Pixies, 1988)

Our minds are magic. Like a prop in an illusionist's sleight of hand, they seem to flit from place to place—now here, now there, now …nowhere. The magic of our minds is often lost in the mundane details of our daily routines, but the remarkable flexibility of our mental lives remains. Our minds may be directed toward the task at hand—they may be “here” as we concentrate on our daily commute or focus on the contents of a meeting or conversation. Our minds may also be “there,” or any place other than the present situation—they may travel to an upcoming vacation, a favorite memory, or even a to-do list as the morning commute turns into a traffic jam or the staff meeting becomes a tedious exercise in endurance. At other times, our minds may go to a third place—neither here nor there, but nowhere. There may be times when our minds are *blank*.

Mind-blanking—when our minds are seemingly “nowhere”—is defined by a lack of conscious awareness. During periods of blankness, the individual is not focally aware of any stimuli, either internal or external. Although this definition may conjure up images of mind-blanking drivers suddenly swerving into oncoming traffic and joggers dropping to the pavement mid-stride, research suggests that conscious awareness is unnecessary for much of human functioning; rather, the vast majority of cognitive processing and behavioral control seems to occur outside of conscious awareness. Countless stimuli are constantly streaming in and around every individual, in the form of both environmental cues and internal trains of thought. Although the majority of these stimuli never reach conscious awareness, they are still perceived—and they may still influence emotions, opinions, decisions, and behavior (e.g., Bargh and Chartrand, 1999) and even lead individuals to form, pursue, and accomplish goals (e.g., Bargh et al., 2001). When stimuli fail to reach conscious awareness and the mind goes blank, the persistence of non-conscious processes may allow people to continue carrying out surprisingly elaborate behaviors, even in the absence of this hallmark of human experience.

However, some mental operations—such as complex information processing, problem solving, and intentional actions—do seem to require conscious awareness (Dehaene and Naccache, 2001). In order for information to enter conscious awareness, it must be attended to. Attention seems to select stimuli from the murky sea of peripherally processed perceptual information and place it before the spotlight of conscious awareness (e.g., Crick and Koch, 1990; Posner, 1994); it selects from several simultaneous possible streams of thought and presents one to conscious awareness, excluding all others (e.g., James, 1907; Baars, 1997; Schooler et al., 2011).

But attention is an unpredictable beast, prone to flights of fancy. It is not constrained to the task at hand, nor even to the present moment. Rather than remaining “here,” in the physically and temporally present perceptual environment, attention—and the conscious awareness it enables—can decouple from perception and allow the mind to transcend the here-and-now of the body's current surroundings (e.g., Wegner, 1997; Mason et al., 2007; Schooler et al., 2011). The decoupling of attention and perception allows the mind to wander—to fill consciousness with ideas related to times unknown, places unseen, and possibilities previously unimagined (as well as other, more practical mental contents—such as what kind of bread to pick up on the way home from work).

Mind-wandering provides evidence that many behaviors can persist unhindered when attention is turned elsewhere. Research suggests that people's minds are separated from their current perceptual environments nearly half the time (Killingsworth and Gilbert, 2010); the mind seems to flit from thought to thought and place to place, stopping in the present environment only when automatic processing cannot handle the task at hand (Mason et al., 2007). For much of human life, the mind is not “here” but “there”—and people do not seem to suffer obvious behavioral deficits on many tasks when attention is decoupled from perception.

If people can function adaptively when attention is elsewhere—when their minds are wandering—then they should also be able to do so when attention is *nowhere*—when their minds are blank. The idea of performing many activities—particularly well-practiced behaviors such as walking, driving, and pretending to listen—while attending to something else seems to be an aspect of everyday life. However, the idea of attention being not just somewhere else, but nowhere at all, may not be as intuitive. A blank mind—a mind in which attention does not call any perceptual input into conscious awareness—seems to be an enigma, eluding introspective insight. There is little to imagine about it beforehand (how can one simulate an absence?), similarly little to remember when it is over (how can one remember nothing?), and any attempts to analyze the contents of one's own blank mind in-the-moment necessarily interrupt the phenomenon of mind-blanking. Although it may make logical sense that people can continue functioning in the absence of conscious awareness, the difficulty of simulating such a state seems to have pushed the blank mind from the realm of possibility—or at least from the focus of empirical study.

However, the blank mind occupies a prevalent role in many lay theories of consciousness. It finds support in the work of William James (1890), who wrote that consciousness could be experienced as continuous even if it contained “interruptions, time-gaps during which the consciousness went out altogether to come into existence again at a later moment.” It is often experienced (or at least pursued) through practices such as meditation (e.g., Campion and Rocco, 2009) and hypnosis (e.g., Holroyd, 2003). And it is the focus of current academic interest, as people trained to periodically ask themselves “Am I conscious now?” often begin to doubt the omnipresence of conscious awareness, and become increasingly less confident that they were conscious in the moments immediately preceding their introspective inquiry (Blackmore, 2002).

The blank mind can also be found in empirical studies—peeking through the cracks of investigations into other questions related to conscious experience. Many studies of attention implicitly deny the existence of mind-blanking by constraining participants such that they cannot report the experience of this mental state; for example, some studies only allow reports of being focused, wandering with awareness, or wandering without awareness (e.g., Smallwood et al., 2008; Sayette et al., 2010). However, reports of mind-blanking have surfaced in studies with less constrained methodologies. One study on task-unrelated thought that allowed participants to report their mental contents in their own words found that people described 18% of task-unrelated mental states as times when they were thinking of “nothing at all” (Schooler et al., 2004). Another study indicates that mind-blanking represents the second-most common form of concentration lapses (Watts and Sharrock, 1985). Studies using descriptive experience sampling, which ping participants throughout the day for self-described reports of mental contents (e.g., Hurlburt and Heavey, 2001, 2008), find that people spontaneously report acting in the absence of conscious thought or awareness (“just doing”). Although the experimental design of many studies has precluded empirical investigations of mind-blanking, those that have allowed for its existence—generally by allowing for unconstrained categorizations or descriptions of mental states—display preliminary evidence in support of the blank mind.

The present work aims to investigate the possibility that there are times when the mind is blank—when conscious awareness is directed neither toward the present perceptual environment nor toward stimuli decoupled from this environment, but nowhere. Mind-blanking shares a fundamental similarity with the well-established mental state of mind-wandering, in that both states represent a decoupling of attention from the current perceptual environment. However, these mental states are also theoretically distinct; whereas the wandering mind represents a state in which attention brings stimuli unrelated to the current task or environment into conscious awareness, the blank mind seems to represent a state in which attention fails to bring any stimuli at all into conscious awareness—the mind is not just elsewhere, but nowhere.

Several empirically supported characteristics of mind-wandering may function as points of comparison with mind-blanking—empirical fingerprints with which to test the relationship between the two mental states, as well as the viability of the blank mind as a distinct mental state. First, mind-wandering is characterized by an ability to recall the prior contents of consciousness, even if the individual was unaware of these contents at the time (e.g., Schooler, 2002; Schooler et al., 2004; Sayette et al., 2009); if people can engage in “mental time travel” and recall content, they should also be able to recall a lack of content—or a blank mind. Second, mind-wandering is associated with impaired performance on tasks that ostensibly require conscious attention—most notably, reading comprehension (e.g., Sayette et al., 2009, 2010; Reichle et al., 2010); mind-blanking may or may not cause similar deficits on this behavioral outcome measure. Third, mind-wandering shows a consistent relationship with meta-awareness, as measured by the ratio of self-caught to probe-caught instances of the wandering mind; this ratio ranges from 1.53:1 (Sayette et al., 2010) to 1.70:1 (Reichle et al., 2010) to 1.95:1 (Sayette et al., 2009). Because mind-blanking is proposed to have a unique relationship with conscious awareness, it may also have a unique relationship with meta-awareness. Fourth, mind-wandering appears to be part of an adaptive attentional cycling system, which causes episodes of mind-wandering to oscillate over time in a pattern anticorrelated with being focused on external stimuli (e.g., Sonuga-Barke and Castellanos, 2007; Smallwood, 2010; Schooler et al., 2011; Mooneyham and Schooler, 2013). Mind-blanking does not seem to fit into the theoretical framework explaining adaptive attentional cycling (a system for efficiently monitoring both internal and external stimuli), and thus may differ from mind-wandering in terms of its relationship with other mental states (i.e., mind-wandering, being focused) over time.

In the present research, we provide evidence for the existence of mind-blanking—a mental state in which attention is decoupled from perception and does not bring any stimuli into conscious awareness. Because mind-wandering, like mind-blanking, represents a decoupling of attention and perception, many of the following experiments make use of techniques pioneered in the mind-wandering literature and focus on comparing and contrasting these seemingly related mental states. Experiment 1 explores phenomenological differences between mind-blanking and mind-wandering by examining whether or not people report both mind-blanking and mind-wandering as experientially distinct mental states. Experiments 2a–2c examine potential differences between mind-blanking and mind-wandering on a behavioral outcome measure: reading comprehension. Experiments 3a–3c address two areas that may reveal differences in the cognitive processes underlying mind-blanking and mind-wandering: the ratio of probe-caught to self-caught episodes (a potential marker of the relationship between each mental state and meta-awareness) and the relationship of each mental state with time. Together, these seven experiments outline the blank mind in terms of four key characteristics of decoupled mental states: phenomenological experience, behavioral outcomes, meta-awareness, and patterns of occurrence over time.

## Experiment 1: phenomenological experience

People often refer to the blank mind as if it represents a unique experience, using phrases such as “I blanked out” or “my mind was a complete blank.” However, these phrases may simply represent imprecise language; they may refer not to the blank mind *per se*, but instead to experiences such as the inability to produce a particular piece of information (as in the tip-of-the-tongue phenomenon; e.g., Brown and McNeill, 1966; Brown, 1991) or the realization that one's attention has turned away from the task at hand (as in mind-wandering; e.g., Wegner, 1997; Schooler et al., 2004; Mason et al., 2007). In this experiment, we investigate whether or not mind-blanking is a phenomenologically distinct mental state by comparing it to another state involving the decoupling of perception and attention: mind-wandering. Does mind-blanking *feel* different than mind-wandering? Are these conceptually unique mental states experienced as such? Or are these mental states identical from the perspective of the mind-blanking and/or mind-wandering individual?

### Method

Twenty-three participants (15 female and 8 male) were recruited from the Harvard University Psychology Study Pool; participants had a mean age of 31.67 years. As in previous studies on mind-wandering (e.g., Sayette et al., 2009, 2010; Reichle et al., 2010), these participants completed a reading task during which they were probed with questions about their mental states; probes occurred randomly, at intervals ranging from 2 to 4 min. In contrast to previous studies of mind-wandering, probes consisted of questions about three different mental states: mind-wandering, mind-blanking, and being focused.

Prior to the experimental task, participants were told that they would be probed throughout the reading task with questions about their immediately preceding mental states. Prior research suggests that people are able to report on prior contents of consciousness, even if they were not aware of them at the time (e.g., Schooler, 2002; Schooler et al., 2004); if people are able to provide post-hoc reports about previously unnoticed mental contents, they should also be able to report an immediately preceding *lack* of mental content.

Participants were then presented with definitions for “mind-wandering,” “mind-blanking,” and “being focused.” These mental states were presented as hypothetical possibilities, as opposed to empirically validated categories of conscious experience. The definition of mind-wandering was borrowed from prior research (Sayette et al., 2009), and read: “at some point during reading, you realize that you have no idea what you just read; furthermore, not only were you not really thinking about the text, you were thinking about something else altogether.” The description of mind-blanking was similar to that for mind-wandering, in that it involved being off-task (that is, decoupling attention from perception); however, the description described a state of complete blankness: “not only were you not really thinking about the text, you were not thinking about anything at all—your mind was a complete blank.” Being focused was described as “paying attention to the text that you were reading.” Together, these mental states encompassed three possible links between attention and conscious awareness: attention could be directed toward the task at hand (“being focused”); attention could be directed toward anything other than the task at hand (“mind-wandering”); or attention could be directed toward nothing at all (“mind-blanking”). After participants had been presented with all three definitions, the differences between mind-blanking and mind-wandering were reiterated, and participants were verbally examined to ensure understanding of all three concepts as well as the differences between a blank mind and a wandering mind.

The experimental task consisted of a 45-min session in which participants were asked to read from the first 6 chapters of Tolstoy's *War and Peace*, a task that has been shown to elicit mind-wandering (Sayette et al., 2009). While reading, participants were randomly probed with questions related to their mental states. Probes consisted of an audible chime paired with a subsequent computer-based questionnaire asking about participants' mental state immediately prior to the chime. These computer screens consisted of two-word probes for each target mental state: mind-wandering (“Mind Wandering?”), mind-blanking (“Mind-Blank?”), and being focused (“Mind Focused?”). For each probe, participants were asked to respond by pressing “y” for yes or “n” for no. Participants received 6 randomly ordered probes of each type, separated by 2- to 4-min. intervals. Self-classified external probes such as these have been used in prior studies assessing the contents of consciousness, and are particularly useful because they do not require individuals to be consciously aware of the contents of their experience while in the midst of this experience (Giambra, 1995; Smallwood and Schooler, 2006). The use of probes in this experiment also reduced the cognitive demands placed on participants; a self-catch methodology requiring participants to consistently monitor for both mind-wandering and mind-blanking may have created a cognitively taxing experimental environment, and this setting may have decreased the accuracy and reliability of the resulting data.

### Results

Participants reported experiencing all three mental states: mind-blanking, mind-wandering, and being focused, with a mean response time for each mental state probe of 3.14 s. Means and standard deviations for each mental state are presented in Table 1. Each mean represents the number of times each mental state was reported out of a total of 6 possible probes. Each percentage represents the percentage of mental state probes that were responded to with an answer of “yes,” indicating that the identified mental state was being experienced immediately prior to the appearance of the probe. Each probe asked about only one mental state at a time; thus, any answer of “no” could indicate that the participant was experiencing either of the two other mental states (e.g., a participant answering “no” to a focus-probe could have been either mind-blanking or mind-wandering).

**Table 1:** **Means and SDs of conscious states in Experiment 1**.

**Mean counts of probed mental states (out of six)**			
**State**	**Mean**	**Percentage**	**Standard deviation**
Mind-blanking	0.87	14.49%	1.49
Mind-wandering	2.96	49.28%	1.72
Focused	3.57	59.42%	1.95

We also tested for correlations between mental states. Mind-wandering was negatively correlated with being focused, *r*_(23)_ = −0.71, *p* < 0.001. Mind-blanking, on the other hand, was uncorrelated with both mind-wandering, *r*_(23)_ = −0.06, *p* = 0.80, and being focused, *r*_(23)_ = −0.26, *p* = 0.24.

### Discussion

These results provide evidence that mind-blanking and mind-wandering are phenomenologically distinct mental states. Participants, informed of potential differences between these two states (but in no way guaranteed that both exist), reported both mind-blanking and mind-wandering. The fact that mind-blanking was reported at all suggests that people are familiar with this experience, even when it is defined in terms of more precise language than may be found in lay descriptions of conscious states (i.e., “I was not thinking about anything at all” as opposed to “I blanked out”). The difference in reported rates of mind-blanking (14.49%) and mind-wandering (49.28%) mirrors previous results related to the incidence of each mental state (mind-blanking: 18%, Schooler et al., 2004; mind-wandering: 46.9%, Killingsworth and Gilbert, 2010), and suggests that people are capable of distinguishing between the two. The lack of correlation between the two states suggests that the two terms were not used interchangeably. If mind-blanking and mind-wandering were positively correlated, this may suggest that people were sloppy categorizers—any off-task mental state was classified as either mind-blanking or mind-wandering, depending on the probe presented. If mind-blanking and mind-wandering were negatively correlated, this may suggest that the two mental states were identical, but were understood differently by different people—some classified any off-task mental state as mind-blanking (and not mind-wandering) whereas others classified any off-task mental state as mind-wandering (and not mind-blanking). The finding that reports of these states were uncorrelated suggests that they are phenomenologically distinct, and that the rate of experiencing one is not necessarily related to the rate of experiencing the other.

Although these results suggest that people experience mind-blanking and mind-wandering as phenomenologically distinct mental states, the differences between these states may be either qualitative or quantitative. Mind-wandering represents a mental state in which attention is decoupled from perception, and directed elsewhere—the mind contains conscious content, this content is simply unconstrained by the current physical or temporal setting. If mind-blanking truly represents a lack of conscious awareness and the persistence of perception in the absence of attention, mind-blanking and mind-wandering seem to be qualitatively different; although both entail a decoupling of perception from attention, conscious awareness persists during mind-wandering but is absent during mind-blanking. However, it may also be that mind-blanking is simply a quantitatively different form of mind-wandering. Recent work suggests that mind-wandering represents a broad category of heterogeneous experiences, each possibly entailing different psychological processes and phenomenological signatures (Smallwood et al., 2013); it may be that one form of mind-wandering consists of directing attention to particularly unremarkable or unmemorable stimuli, thus creating a mental state that is easily categorized as a blank mind. Although participants reported mind-blanking and mind-wandering as phenomenologically distinct experiences, it is possible that apparent qualitative differences between these mental states may in fact reflect quantitative differences related to the memorability of mental contents present during decoupled mental states.

People's demonstrated ability to perform “mental time travel”—that is, to analyze the contents of their own consciousness after-the-fact—suggests that mind-blanking may be qualitatively, not quantitatively, different from mind-wandering. In the case of mind-wandering, participants are able to report what they were previously thinking about, even though they were ostensibly unaware of it at the time (Schooler, 2002; Schooler et al., 2004). If people are able to report the contents of their thoughts after-the-fact, then they should also be able to report the absence of thought—that is, the experience of mind-blanking. Research suggests that even unattended and irrelevant information can be explicitly remembered after-the-fact (e.g., Hoffman et al., 2011), suggesting that information does not need to be particularly engaging in order to be reported post-hoc, and that reports of blankness are unlikely to simply reflect an inability to recall mundane or monotonous thoughts.

However, it may be more difficult to recall certain types of thoughts after-the-fact, even if this recollection is possible. Perhaps some thoughts—those that are irrelevant or uninteresting—are less memorable to start with, and may fade from memory more quickly than others. Mind-wandering directed toward these sorts of particularly forgettable thoughts might be experienced or remembered as mind-blanking; reports of mind-blanking would still reflect the occurrence of a phenomenologically distinct state, but this distinction from mind-wandering would be related to an inability to remember conscious contents, rather than a true lack of content. The current experiment used probes in order to combat the potential issue of miscategorization based on memory failure. Mental state probes minimize memory requirements by asking participants to report mental states as close to in-the-moment as possible; probes interrupt mental states as they are occurring, and simply ask participants to indicate their conscious contents in the instant before the probe. In this experiment, mean response time to the specific, two-word probes was only 3.14 s; within this short span, participants attended to the probe, read the probe, assessed their mental state, and responded. People's general ability to explicitly recall previously unnoticed mental contents, the immediate nature of mental state probes in general, and the short response time to the probes used in this experiments suggest that reports of mind-blanking—both here and elsewhere—may not simply reflect miscategorizations due to memory failure. Rather, mind-blanking and mind-wandering may represent two qualitatively different, but related, mental states.

## Experiments 2a– 2c: Behavioral Outcomes

Experiment 1 focuses on internal experience, suggesting that people experience mind-blanking and mind-wandering as two phenomenologically distinct mental states. In these experiments, we turn our focus from the internal experience of mind-blanking to external, behavioral outcomes associated with this mental state. Specifically, we investigate the effect of mind-blanking on reading comprehension, a common marker of the ability to attend to and process stimuli present in the immediate environment.

Experiments 2a, 2b, and 2c are based on the “zoning-out during reading” task (e.g., Schooler et al., 2004), which is often used to examine the effect of mind-wandering on task-related performance. This task consists of assessing participants' mental states while they read a block of dense text. After the reading period is finished, participants complete a reading comprehension test. Correlations between mental states (generally, mind-wandering) and reading comprehension are then computed in order to assess the impact of these mental states on performance. Specifics of the experimental design for each experiment are presented in Table 2.

**Table 2:** **Experimental design of Experiments 2a, 2b, and 2c**.

**Experiment**	**Participant details**	**Reading material**	**Reading time**	**Reading speed**	**Notes**
2a	N = 27	War and peace	20 min	Own pace	Mental state reports were double-checked
	15 Female				
	M_age_ = 25.38				
2b	N = 56	Anna Karenina	20 min	Own pace	Participants described contents of preceding mental states
	33 Female				
	M_age_ = 27.90				
2c	N = 75	Anna Karenina	Unlimited	Own pace, auto scrolling	None
	36 Female				
	M_age_ = 29.85				

### Experiments 2a and 2b

#### Method

These experiments followed nearly identical methodology. However, they used different methods to ensure the accuracy of self-reported mind-blanking in each experiment. In both experiments, participants were recruited from the Harvard University Psychology Study Pool. Upon entering the lab, participants were asked if they had ever read a piece of classic literature—either *War and Peace* (2a) or *Anna Karenina* (2b); if participants indicated that they were familiar with the relevant work, they were redirected to another study. The remaining participants were then informed that they would have 20 min to read a selection from the book, and that they would be tested on the material once the 20 min had passed.

Next, participants were given definitions of both mind-blanking and mind-wandering. These definitions were identical to those provided in Experiment 1. However, an additional distinction related to the phenomenological experiences reported by participants in Experiment 1 was added for this experiment: “The only difference between mind-wandering and mind-blanking is that in mind-wandering you're thinking about something else, and in mind-blanking you aren't.”

These experiments used self-catch, as opposed to probe-catch, measures. In order to reduce latency between the experience of mind-blanking or mind-wandering and reports of that mental state, self-reports followed a two-step procedure. First, participants pressed a button if they realized that they were no longer attending to the reading. Second, participants pressed one of two additional buttons to indicate whether their attentional lapse had been due to mind-blanking or mind-wandering.

Participants in both experiments were required to confirm their mental state reports. In Experiment 2a, they were asked to confirm their initial decision by answering the question “Are you sure?” on a dichotomous yes/no scale. This confirmation check was designed to encourage participants to engage in “mental time travel” and ensure that their minds were empty (if they reported mind-blanking) or contentful (if they reported mind-wandering). In Experiment 2b, participants who had just indicated that their minds were either blank or wandering were subsequently asked to provide written information in free response form about their prior mental state, including any memories they had of thoughts during this state and when or how they realized that their thoughts had become divorced from the task at hand. This confirmation check was designed to both discourage participants from reporting artificially high levels of either mental state and gather information about lay conceptualizations of mind-blanking and mind-wandering.

After receiving all instructions, participants were quizzed on the definitions of mind-blanking and mind-wandering, reminded of the procedures for reporting and confirming/describing mental states, and given the appropriate text. After 20 min, participants were asked to stop reading and were instructed to complete a 10-item test of reading comprehension related to the material they had just seen.

#### Results

Participants in Experiments 2a and 2b reported both mind-blanking and mind-wandering. Confirmation checks from each experiment provided additional evidence that these mental states are phenomenologically distinct, and that participants were confident about their ability to distinguish between the two. In Experiment 2a, the majority of initial reports of both mind-blanking and mind-wandering were doubly confirmed—that is, participants reported being sure that their minds were blank and not wandering (or wandering and not blank); see Table 3 for means and standard deviations of each mental state in this experiment, as well as the percentage of each mental state that was doubly confirmed. Only doubly confirmed reports of each mental state are used for all further analyses of results from Experiment 2a.

**Table 3:** **Means and SDs of conscious states in Experiment 2a**.

**Mean counts of self-caught mental states (over 20 min reading period)**		
**State**	**Mean**	**Standard deviation**
Mind-blanking	3.26	4.40
Mind-blanking, confirmed	2.96	4.43
Mind-blanking, % confirmed	81.3%	0.33
Mind-wandering	4.78	5.21
Mind-wandering, confirmed	4.67	5.22
Mind-wandering, % confirmed	96.6%	0.09

In Experiment 2b, spontaneous descriptions of each mental state suggested that participants conceived of the two states differently. For example, one participant described an instance of mind-blanking by saying “My mind was blank. I realized this when I was just staring blankly at a sentence and not reading it. I think I only stared at the sentence for a few seconds before I snapped out of it,” and an instance of mind-wandering by saying “My mind was wandering. I realized I was wandering when I was lost in what I was reading. I remember thinking about something I have to do later today.” Another participant described a blanking experience when she “found myself staring at the same word but I don't remember reading it” and a wandering experience as “found myself reading but not knowing what I was reading.” Still other participants seem to indicate that the boundary between mind-blanking and mind-wandering—though clear—may be easily crossed; for example “at first my mind started to wander intentionally as I imagined what it was like to live in the household that was being described but I was able to read one or two more sentences while still keeping feeling in the front of my mind but then went blank.” All reports of both mind-blanking and mind-wandering are used for further analysis of results from Experiment 2b.

As in Experiment 1, reports of mind-blanking and mind-wandering were uncorrelated in both Experiment 2a, *r*_(27)_ = 0.172, *p* = 0.39, and Experiment 2b, *r*_(56)_ = −0.173, *p* = 0.20.

In line with previous research on mind-wandering, increased incidence of mind-wandering was correlated with decreased performance on the reading comprehension test in both Experiment 2a, *r*_(26)_ = −0.40, *p* = 0.04, *r*^2^ = 0.16 and Experiment 2b, *r*_(56)_ = −0.41, *p* < 0.01, *r*^2^ = 0.17. Mind-blanking, on the other hand, did not affect scores on the reading comprehension test in either Experiment 2a, *r*_(26)_ = −0.005, *p* = 0.98, or Experiment 2b, *r*_(56)_ = 0.06, *p* = 0.68. A follow-up stepwise multiple regression with mind-wandering and mind-blanking as possible predictors of test performance confirmed that mind-wandering accounted for all differences on performance in Experiment 2a: full regression *F*_(1, 25)_ = 4.56, *p* = 0.04; β_wander_ = −0.40, *t* = −2.14, *p* = 0.04; β_blank_ = *ns*. A second stepwise multiple regression with the same factors for Experiment 2b confirmed that mind-wandering accounted for all difference in test performance in this experiment: full regression *F*_(1, 54)_ = 10.74, *p* < 0.01; β_wander_ = −0.41, *t* = −3.28, *p* < 0.01; β_blank_ = *ns*.

#### Discussion

Results from these experiments both corroborate the findings of Experiment 1—that mind-blanking and mind-wandering are phenomenologically distinct experiences—and provide evidence that these mental states are associated with different behavioral outcomes. As in Experiment 1, participants reported both mind-blanking and mind-wandering, and these two mental states were not correlated with each other. As an expansion of Experiment 1, participants were asked to re-assess their initial reports of both mind-blanking and mind-wandering, and either confirm their initial reports (Experiment 2a) or describe their preceding mental contents (or lack thereof, Experiment 2b). Although participants in Experiment 2a seemed to be more confident about reports of mind-wandering (96.6% confirmed) than about reports of mind-blanking (81.3% confirmed), both mental states were confirmed more often than not. Free response descriptions from Experiment 2b provide further evidence that people both experience and describe these mental states differently, and are capable of distinguishing between them.

The possibility of blurred boundaries between mind-blanking and mind-wandering, as reported in Experiment 2b, may provide insight into the relatively low confirmation rate for mind-blanking, relative to mind-wandering, in Experiment 2a. Although these mental states are distinct, they may often fade from one to the other (e.g., “at first my mind started to wander … but then went blank”). When confirming a wandering mind, one must only confirm that his or her thoughts were unrelated to the immediate situation. However, when confirming a blank mind, one must confirm that he or she had no thoughts whatsoever. If these two states are often fading back and forth—from mind-blanking to mind-wandering—memories of stray thoughts may taint confirmations of mind-blanking and introduce uncertainty into initial mental state judgments. Just like a drop of poison contaminates a glass of water but a drop of water does nothing to purify a glass of poison, a single remembered thought may taint categorizations of the blank mind whereas moments of blankness may not affect categorizations of mind-wandering.

Further data from this experiment indicate that mind-blanking and mind-wandering are associated with different behavioral outcomes. Specifically, mind-wandering seems to impair reading comprehension, whereas mind-blanking does not. Evidence from previous mind-wandering studies suggests that people's eyes continue to move across the page while their minds wander (e.g., Schooler et al., 2004; Reichle et al., 2010). This suggests that they are unaware that their attention has drifted elsewhere, and provides a prime example of the decoupling of perception and attention; perception (and the appearance of conscious processing) persists in the absence of attention (and a corresponding absence of conscious awareness of the unattended text). Mind-wandering “readers” may continue to maintain the appearance of reading, while failing to process information contained within the text—an irony that becomes apparent when these individuals are tested on the material that their eyes scanned, but their minds failed to process. Although many behaviors do not seem to require attention or conscious awareness, the deleterious effects of mind-wandering on reading comprehension suggests that processing dense text does require conscious awareness.

Mind-blanking, too, seems to represent a decoupling of perception from attention; however, this state does not seem to impair reading comprehension. At least three possible explanations could account for this lack of connection between mind-blanking—a mental state that ostensibly prevents the individual from consciously processing stimuli—and reading comprehension. First, it may be that both mind-blankers and mind-wanderers appear to attend to the reading task (e.g., by moving their eyes across the page) while their attention is elsewhere (or, in the case of mind-blanking, nowhere). If this is the case, the only difference between the two states may be the typical duration of each mental state; mind-wandering, an immersive experience, may continue for pages at a time whereas mind-blanking, a state defined by the lack of experience, may occur only briefly and intermittently—unlike mind-wandering, there is no narrative arc or constant concern to extend the length of periods of mind-blanking. If this is the case, mind-blanking may result in less missed text relative to mind-wandering, and may consequently have less deleterious effects on tasks that require conscious awareness (such as reading comprehension).

A second, and related, possibility is that the less immersive experience of mind-blanking is more easily interrupted by a task that requires conscious attention. During mind-wandering, attention may be directed to an engaging or important stimulus or train of thought; during mind-blanking, on the other hand, attention is directed nowhere. Thus, mind-blankers may be more likely than mind-wanderers to “snap out of it” and return to the task at hand when this task requires attention. Unlike mind-wanders, mind-blankers have no conflict over which stream of information is more worthy of their attention.

The first two explanations suggest that mind-blanking produces less interference than mind-wandering for tasks that require conscious attention; mind-blanking is either intermittent and short-lived, or easily interrupted by stimuli requiring conscious attention. Conversely, the third explanation suggests that mind-blanking may interfere *more* than mind-wandering for tasks that require conscious attention. It may be that mind-blanking entails not just a lack of conscious awareness, but also a corresponding failure to display the hallmarks of conscious awareness (in this case, moving one's eyes across a page of text without actually attending to this information). Basic activities (e.g., walking, driving, and other well-practiced behaviors) may persist while mind-blanking, but actions that require conscious attention may not only fail to occur, but also fail to *appear* as if they are occurring. In the case of reading, this would cause people to stop moving their eyes across the page when their minds go blank. When their minds return from this state, they may simply pick back up where they left-off—and avoid suffering the deficits in reading comprehension that seem to come from maintaining the appearance of attention without actually attending to the task at hand (in this case, continuing to move one's eyes across a page without processing the information).

### Experiment 2c

Although both mind-blanking and mind-wandering seem to represent a decoupling of perception and attention, previous results (Experiments 2a, 2b) indicate that only mind-wandering has a deleterious effect on processes requiring conscious attention—specifically, reading comprehension. The present experiment was designed to investigate potential explanations for this apparent difference between mind-blanking and mind-wandering on behavioral outcomes. Specifically, we wanted to investigate the third possibility outlined above: that when mind-blanking, people not only stop attending to the current perceptual environment, but also fail to appear as if they are doing so (at least when the demands of the environment require conscious attention). In order to test this explanation for the differing effects of mind-blanking and mind-wandering on performance, we examined whether forcing people to read auto-scrolling text—text that they could not simply pick back up where they left-off—would eliminate the difference between each decoupled mental state's effects on behavioral performance as found in Experiments 2a and 2b.

#### Method

Seventy-five participants (36 female and 39 male) were recruited from the Harvard University Psychology Study Pool; participants had a mean age of 29.85 years. The experimental design was similar to that used in Experiments 2a and 2b, with the exception that participants were divided into two reading conditions. Participants in both conditions were assigned to read a passage from *Anna Karenina*, but those in the “self-paced” condition could read at their own pace, whereas those in the “auto-scrolling” condition read text as it automatically scrolled across a computer screen (at a speed chosen by each participant to be neither too slow nor too fast). If different relationships between each mental state (mind-blanking, mind-wandering) and reading comprehension are due to mind-blankers being more likely than mind-wanderers to pick up reading where they left-off, auto-scrolling text should prevent this strategy—and eliminate the apparent differences in behavioral outcomes associated with these mental states.

Participants were provided with the definitions of mind-blanking and mind-wandering used in all other experiments. Once it was clear that they understood these definitions, they were administered the reading task and told that there would be a quiz once this task was finished. Participants in the “self-paced” condition were allowed as much time as they needed to finish the passage, whereas those in the “auto-scrolling” condition were required to read the text as it scrolled by at their pre-selected speed. As in Experiments 2a and 2b, participants were instructed to press a button any time they noticed that their attention was no longer focused on the task, then select whether they had been blanking or wandering. After participants finished the reading task, they were administered a reading comprehension test.

#### Results

As in all other experiments, participants reported both mind-blanking and mind-wandering, and these reports were not correlated with each other, *r*_(75)_ = 0.105, *p* = 0.37.

For participants in the self-paced condition, which mirrored the experimental design of Experiments 2a and 2b, mind-wandering was negatively correlated with performance on a reading comprehension text, *r*_(38)_ = −0.463, *p* < 0.01, *r*^2^ = 0.21; mind-blanking, again as in Experiments 2a and 2b, was not, *r*_(38)_ < 0.001, *p* = 1.00. A follow-up stepwise multiple regression with mind-wandering and mind-blanking as possible predictors of test performance confirmed that mind-wandering accounted for all differences on performance: full regression *F*_(1, 37)_ = 9.81, *p* < 0.01; β_wander_ = −0.46, *t* = −3.13, *p* < 0.01; β_blank_ = *ns*.

**Figure 1:**
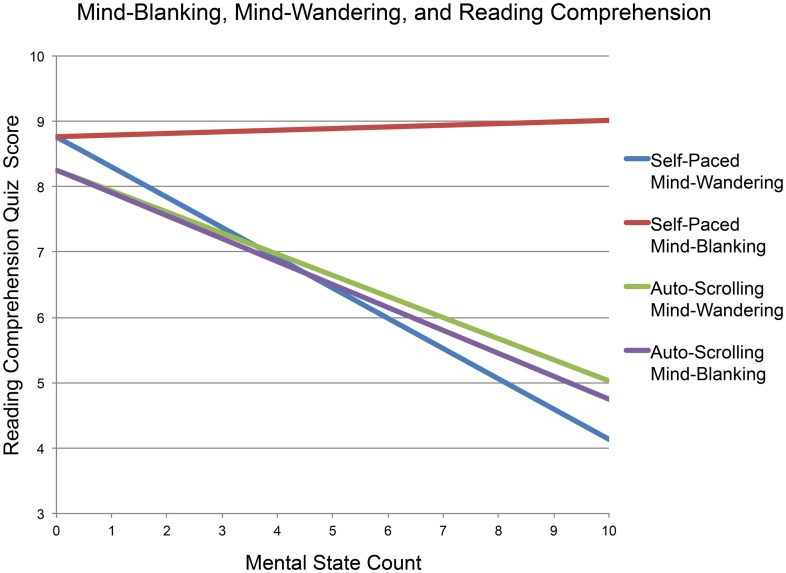
**Relationship between mind-blanking, mind-wandering, and reading comprehension, under both self-paced and auto-scrolling conditions in Experiment 2c**.

However, participants in the auto-scrolling condition displayed a different pattern of results. As in the self-paced condition, mind-wandering was negatively correlated with performance on a reading comprehension test, *r*_(37)_ = −0.357, *p* = 0.03, *r*^2^ = 0.13. However, unlike in all previous experiments, mind-blanking in the auto-scrolling condition was also associated with impaired performance on a reading comprehension test, *r*_(37)_ = −0.383, *p* = 0.02, *r*^2^ = 0.15. A follow-up stepwise multiple regression with mind-wandering and mind-blanking as possible predictors of test performance confirmed that both mind-wandering and mind-blanking influenced test performance: full regression *F*_(1, 36)_ = 5.66, *p* < 0.01; β_wander_ = −0.35, *t* = −2.35, *p* = 0.03; β_blank_ = −0.32, *t* = −2.16, *p* = 0.04. A comparison of Fisher's (1915) z-scores for correlations between mind-blanking and test performance in the self-paced and auto-scrolling conditions confirmed that mind-blanking was more related to test performance in the auto-scrolling condition than in the self-paced condition, *z* = 1.68, *p* = 0.04. Plotted regression equations in Figure 1 display the effect of each mental state (mind-wandering, mind-blanking) on reading comprehension, when incidence of the other mental state is held constant.

#### Discussion

Results from this experiment provide evidence that apparent differences in the effects of mind-blanking and mind-wandering on behavioral outcomes requiring conscious attention (e.g., reading comprehension) may be due to differences in the persistence of processes related to the appearance of conscious attention; these processes seem to be prevalent while mind-wandering, but less prevalent (or perhaps non-existent) while mind-blanking. When people's minds wander, their eyes continue to move across the page and, upon returning from mind-wandering, they may continue reading from where their eyes left off—and not from where they stopped attending to the text; as a result, they may miss large portions of information and suffer deficits on tests of reading comprehension. When people's minds go blank, however, it appears that they momentarily pause not just the act of attending to external information, but also the appearance of doing so. When these people return from mind-blanking, they may pick up from where they stopped paying attention—a position that, unlike in mind-wandering, coincides with where they appeared to stop engaging with the external world. Results from this experiment suggest that mind-blanking may be less likely than mind-wandering to result in glossing over important information, and may thus be less likely to result in deficits on tests of behaviors dependent on conscious awareness (e.g., reading comprehension).

These results suggest that mind-blanking represents a more complete break from conscious awareness than does mind-wandering; whereas mind-wanderers let their attention drift elsewhere while they appear to attend to the task at hand, mind-blanking individuals suspend both paying attention to the external task and appearing to do so. Both states seem to allow for the processing of information that does not require conscious attention (that is, processing that can occur automatically based solely on perceptual input that need not reach conscious awareness). However, neither state allows people to process external information that *does* require conscious attention. Individuals in a state of mind-wandering exhibit the external signs of attending to this information, even though their attention is truly directed elsewhere. Mind-blanking individuals, on the other hand, do not exhibit these external hallmarks of attention; while in this state, their lack of conscious awareness does not seem to be disguised by appearances of attention—deceptive appearances which, in the case of operations actually requiring conscious attention (such as reading comprehension), may be associated with negative behavioral outcomes.

## Experiments 3a– 3c: meta-awareness and time

Experiments 1 and 2 provide evidence that mind-blanking has a distinct psychological signature, both in terms of phenomenology (what mind-blanking “feels” like to the internal experiencer) and behavioral outcomes (effects of mind-blanking that may be apparent to outside observers). The following experiments analyze additional characteristics of the blank mind that may often elude detection, both internally and externally: the relationship of mind-blanking with meta-awareness, and the occurrence of mind-blanking over time. These characteristics have been explored previously in the domain of mind-wandering, and may provide additional insight into the cognitive processes underlying mind-blanking. The following experiments also provide further confirmation of several results presented in Experiments 1 and 2, including the patterns of correlations between mind-blanking and mind-wandering and the behavioral outcomes associated with each mental state.

### Method

Experiments 3a, 3b, and 3c were designed to complement each other. Like Experiments 2a–2c, each uses a methodology based on the “zoning out while reading” task (e.g., Sayette et al., 2009, 2010; Reichle et al., 2010). However, each experiment includes minor tweaks—related, for example, to the mental states assessed, the method used to measure these mental states, and the organization of the reading period. Specifics of experimental design for each experiment are presented in Table 4; methodological details and specifics of analysis for each experiment—as well as additional data collected in Experiment 1—are provided in the Appendix. Together, these closely related experiments allow for deeper insight into mind-blanking without imposing unnecessary cognitive demands on participants. Due to their interrelated nature, the results and implications of these experiments will be discussed together.

**Table 4:** **Experimental design of Experiments 3a, 3b, and 3c**.

**Experiment**	**Participant details**	**Location**	**Reading time**	**Mental states assessed**	**Measurement method**
3a	*N* = 27	Lab	20 min	Blanking, wandering	Self-Catch
	13 Female				
	*M*_age_ = 28.61				
3b	*N* = 108	Home	Two blocks, 6 min each	Blanking	Self-Catch
	74 Female				
	*M*_age_ = 33.69				
3c	*N* = 143	Home	30 min	Blanking	Probes, Self-Catch
	82 Female				
	*M*_age_ = 33.30				

These experiments followed the basic protocol of the standard “zoning-out during reading” task (e.g., Schooler et al., 2004). Each participant was assigned to read a block of dense text (in this case, *War and Peace*). Prior to reading, participants in all experiments received descriptions of both mind-wandering and mind-blanking (see descriptions in Experiment 1). Participants were required to verbally confirm understanding of each concept. Participants then began the reading task; after the reading task was finished, participants completed a reading comprehension test (Experiment 1, Experiment 3a). Finally, participants filled out a demographic questionnaire and were debriefed.

Mental states while reading were assessed using both probe and self-catch measures, both in the lab (1, 3a) and at home (3b, 3c). Data for home sessions were collected using Amazon mTurk, which provides a more diverse sample than lab-based methods while maintaining equivalent levels of reliability (Buhrmester et al., 2011). In experiments using probes, probes were randomly administered at time intervals ranging from 2 to 4 min; this replicates previous mind-wandering research using the “zoning out while reading” task (e.g., Sayette et al., 2009, 2010; Reichle et al., 2010). In experiments using self-catch methods, participants were asked to press the spacebar on a computer whenever they noticed they were off-task (3a) or blank (3b, 3c); if asked to report being off-task (3a), they subsequently categorized their prior mental state as either blanking or wandering.

### Results and discussion

#### Descriptive statistics and phenomenology

If mind-blanking represents a phenomenologically distinct mental state, people should report experiencing both mind-blanking and the seemingly related mental state of mind-wandering (as in Experiments 1 and 2). Participants reported both mind-blanking and mind-wandering, through self-catch measures (3a, 3b, 3c) and in response to probes (1, 3c). A paired-samples *t*-test revealed that self-catch reports of each mental state did not differ; participants reported both mind-blanking (*M* = 3.26, *SD* = 4.4) and mind-wandering (*M* = 4.78, *SD* = 5.21) at similar rates, *t*_(26)_ = 1.27, *p* = 0.215 (3a). However, a second-paired-samples *t*-test revealed that probes were more likely to catch mind-wandering (*M* = 2.96, *SD* = 1.72) than mind-blanking (*M* = 0.87, *SD* = 1.49), *t*_(22)_ = 4.29, *p* < 0.001 (1). This discrepancy suggests that the measurement technique used to assess each mental state may produce different estimates of that state's occurrence. In particular, it may be that probe-catch methods underestimate the incidence of mind-blanking; although probe-catch methods indicated lower levels of each mental state than self-catch methods did, this discrepancy was more pronounced for mind-blanking (*M*_self_ = 3.26, *M*_probe_ = 0.87; *M*_diff_ = 2.39) than for mind-wandering (*M*_self_ = 4.78, *M*_probe_ = 2.98; *M*_diff_ = 1.82).

#### Meta-awareness

Self- and probe-caught reports of mental states can be used to assess the relationship between this mental state and meta-awareness (Schooler et al., 2011). The discrepancy between self- and probe-caught instances of mind-blanking suggests that this mental state is more likely to be self-caught than caught by probes, and the apparently larger discrepancy between these two measurement methods for mind-blanking relative to mind-wandering suggests that these two mental states may have different relationships with meta-awareness—specifically, meta-awareness may be more likely to interrupt mind-blanking than mind-wandering. A paired-samples *t*-test comparing the output of each measurement technique when administered during the same experimental period revealed that mind-blanking was significantly more likely to be caught by self-catch methods (*M* = 5.01, *SD* = 6.62) than by probes (*M* = 1.23, *SD* = 1.54), at a ratio of 4.07:1 (3c). Despite our use of an identical methodology, this ratio differs markedly from ratios of self- to probe-caught mind-wandering identified in previous research, which range from 1.53:1 (Sayette et al., 2010) to 1.70:1 (Reichle et al., 2010) to 1.95:1 (Sayette et al., 2009). These results suggest that mind-blanking may be more likely than mind-wandering to be self-caught before it can be caught by an external probe.

The contrasting relationships between these mental states and meta-awareness may be related to potential differences in the adaptive qualities of each mental state. Although evidence suggests that mind-wandering may serve various adaptive functions (e.g., Baars, 2010; Baird et al., 2011), it is unclear whether or not mind-blanking is similarly adaptive. If mind-blanking is less adaptive than mind-wandering, the mind may monitor and correct for the blank mind more than the wandering mind, causing mind-blankers to “snap out of it” before they can be confronted by a probe. The propensity of mind-blankers to catch themselves before they are confronted by probes could also be due to basic properties of mind-blanking; it may be that instances of mind-blanking are typically shorter than mind-wandering episodes, even without the interference of meta-awareness. Although the reason for the apparent difference between mind-blanking and mind-wandering in terms of their relationships with meta-awareness is unclear—it may be due to properties of meta-awareness, properties of the blank mind itself, or still other phenomena—the existence of this difference suggests that mind-blanking and mind-wandering, though closely related, may be associated with different underlying cognitive processes.

#### Time

The world abounds with potentially relevant stimuli, and the ability to attend to as much of this information as possible may enable individuals to function in a maximally adaptive fashion. One way of attending to multiple stimuli within the confounds of an attentional system that seems to allow only one target of conscious awareness at a time (e.g., James, 1907; Baars, 1997) may be to engage in attentional cycling (e.g., Schooler et al., 2011; Mooneyham and Schooler, 2013), a system that entails rhythmically shifting one's attentional focus between multiple potentially relevant streams of information. Because both being focused and mind-wandering require attention (directed either toward the task at hand or toward some stimulus-independent thought) and generally entail conscious awareness, the existence of an adaptive attentional cycling system would suggest that these mental states should be negatively correlated over time—increases in one state at any given time should be associated with decreases in another. If mind-blanking, which does not entail conscious awareness, is not a part of this system of attentional cycling, then it should not be correlated with either mind-wandering or being focused over time.

**Figure 2:**
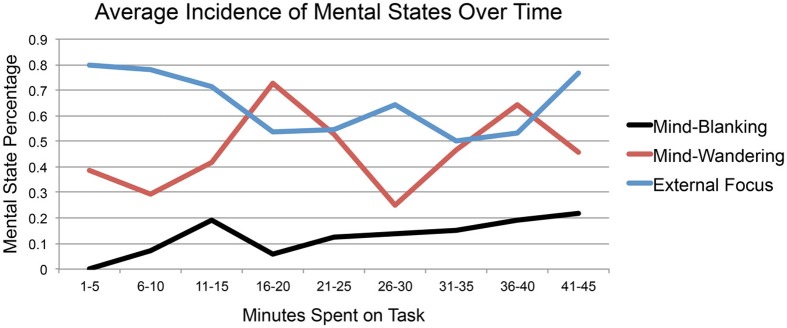
**Average incidence of mind-blanking, mind-wandering, and focusing on external stimuli in Experiment 1**.

Reports of mind-wandering over time seem to support the attentional cycling hypothesis (e.g., Fox et al., 2005; Sonuga-Barke and Castellanos, 2007; Smallwood, 2010; Schooler et al., 2011). Mind-wandering showed a significant cubic trend over time *F*_(1, 26)_ = 12.14, *p* < 0.01 (3a) and was anticorrelated with being focused over time, *r*_(7)_ = −0.663, *p* = 0.05 (1), suggesting that the mental states of mind-wandering and focusing on external stimuli follow alternating cubic trends. See Figure 2 for average incidences of mind-wandering and external focus, as well as mind-blanking, over the course of a 45-min reading task (1).

Mind-blanking, on the other hand, does not seem to be a part of this attentional cycle; rather, occurrences of mind-blanking appear to stay somewhat steady over time. Probe-caught reports of mind-blanking increased over time, *r*_(7)_ = 0.80, *p* < 0.01 (1), and self-caught reports of mind-blanking decreased over time, *F*_(1, 26)_ = 6.84, *p* = 0.02 (3a). When probe- and self-catch measures were administered together together, the ratio of probe- to self-caught blanking increased with the passage of time, *t*_(62)_ = 6.83, *p* < 0.001, with *r*_mean_ = 0.39 (3c). Together, these data suggest that the incidence of mind-blanking *per se* may not fluctuate considerably over time, but that the relative incidence of probe- and self-caught mind-blanking changes as task duration increases, such that blanking is relatively more likely to be caught by probes than by the self as people continue to engage in a given task.

This pattern of results suggests that the relationship of mind-blanking and meta-awareness changes over time, and that people become less aware of mind-blanking as time goes on unless they are forced to introspect upon their mental states by an external force (e.g., a probe). Results from Experiment 3b suggest that this apparent shift in the relationship between mind-blanking and meta-awareness is not simply an artifact of experimental design. In this experiment, participants reported self-caught mind-blanking in two back-to-back reading periods; a decreasing trend of reports over time occurred within each reading period, *F*_time(1, 212)_ = 45.14, *p* < 0.001, but total mind-blanking did not differ between the two periods, *F*_period(1, 212)_ = 1.69, *p* = 0.20 (3b). If decreasing reports of self-caught mind-blanking over time were simply due to experimental artifacts such as hypervigilance when first monitoring for this mental state—which could lead people to over-report the blank mind early on, before becoming comfortable with the experimental paradigm—one would expect decreases in reports of mind-blanking over time to only occur during each participant's first experience with self-monitoring mental states; however, these results indicate that such a habituation or acclimatization effect does not occur, as reports of mind-blanking exhibited the same pattern over time even when tested in two back-to-back experimental sessions. It seems that the apparent shift between self-caught and probe-caught blanking over experimental periods is not an artifact of the experimental environment, but is due to the nature of the relationship between mind-blanking and meta-awareness.

Taken together, these analyses of the relationship between mental states—mind-blanking, mind-wandering, and attending to external stimuli—and time suggest that mind-blanking and mind-wandering may occupy two different roles within the taxonomy of human attention. Mind-wandering, or stimulus-independent thought, may be part of an adaptive attentional cycling process allowing organisms to monitor multiple relevant streams of information. Mind-blanking, on the other hand, does not seem to be a part of this cyclical system; rather, it seems to occur less frequently than either mind-wandering or focusing on external stimuli (see Table 1, as well as descriptive statistics from Experiments 3a–3c), and at a relatively consistent rate over time. Mind-wandering and being focused are negatively correlated over time, suggesting that attending to a stimulus-independent train of thought precludes attending to the present perceptual environment; this necessitates that any organism wishing to monitor multiple streams of information switch attention between internal and external focal points. Mind-blanking, on the other hand, is not correlated with either mind-wandering or being focused over time, suggesting that the blank mind does not interfere with this cycle.

These differences between mind-blanking and other mental states in terms of occurrence over time may be related to the fundamental nature of the blank mind. Although mind-blanking, by definition, precludes the individual from attending to any information, the prevalence of this mental state does not seem to interfere with the occurrence of other mental states. Mind-blanking may represent a “blip” in the attentional system, an unintended lack of conscious awareness that occurs when there are momentary gaps in the mind's ability to locate stimuli capable of breaching the attentional threshold. Or the blank mind may represent a baseline mental state—an evolutionarily prior system of functioning similar to those employed by creatures ostensibly lacking conscious awareness, upon which all conscious states are built.

#### Correlations

Further results from this series of experiments corroborate the findings of Experiments 1 and 2. As in these experiments, reports of mind-blanking and mind-wandering were uncorrelated with each other, *r*_(27)_ = 0.17 *p* = 0.39 (3a). As in Experiment 1, this lack of correlation between mind-blanking and mind-wandering suggests that these mental states are associated with distinct phenomenological experiences, and that people are capable of reliably categorizing these experiences.

#### Test performance

These experiments also confirm the performance-related findings presented in Experiment 2. High levels of mind-wandering were associated with poor scores on a reading comprehension test, both when assessed by probes, *r*_(23)_ = −0.50, *p* = 0.02, *r*^2^ = 0.25 (1), and when assessed using self-report methods, *r*_(27)_ = −0.40, *p* = 0.04, *r*^2^ = 0.16 (3a). As in Experiment 2, mind-blanking did not impair reading comprehension, whether assessed through probes, *r*_(23)_ = −0.021, *p* = 0.92 (1), or through self-catch methods, *r*_(27)_ = −0.005, *p* = 0.98 (3a).

### Discussion

The results of Experiments 3a, 3b, and 3c, along with additional results from Experiment 1, provide insight into the relationship of mind-blanking with meta-awareness and time, and corroborate previous evidence related to the phenomenology and behavioral outcomes of mind-blanking. These results suggest that mind-blanking and mind-wandering have distinct cognitive signatures, in addition to their differing phenomenological properties and associated behavioral outcomes. Mind-blanking seems more susceptible than mind-wandering to interruption by meta-awareness. Perhaps more interestingly, mind-wandering seems to play an important role in an adaptive attentional cycling system that allows the individual to attend to multiple potentially relevant streams of information. Mind-blanking, on the other hand, does not appear to be a part of this attentional cycling system; unlike mind-wandering, the occurrence of mind-blanking does not seem to entail direct trade-offs related to other mental states. This relationship with other mental states over time suggests that mind-blanking may be either relatively unimportant—a mere interruption in the link between perception, attention, and consciousness—or fundamentally important—a foundational mental state upon which this link is built.

## General discussion

Seven experiments provide evidence supporting the existence of the blank mind as a distinct mental state with a unique psychological signature. These experiments distinguish mind-blanking from the seemingly similar mental state of mind-wandering by indicating that these mental states correspond with distinguishable phenomenological experiences (Experiment 1), are associated with different behavioral outcomes (Experiments 2a, 2b, 2c), display dissimilar relationships with meta-awareness (Experiments 1, 3a, 3c), and occupy non-overlapping roles within the system of attentional cycling (Experiments 1, 3a, 3b, 3c); see Tables 5–7 for meta-analyses of these results. By differentiating mind-blanking from other mental states—including mind-wandering—these experiments provide preliminary evidence that the blank mind *exists*. This possibility—that there are times when the mind is neither here nor there, but nowhere—has largely been ignored by empirical investigations of consciousness and attention. The present research opens the door for an expanded taxonomy of mental states, one that allows not just for states defined by stimulus-dependent or stimulus-independent conscious contents, but also for a state defined by a lack of conscious content altogether.

**Table 5:** **Meta-analysis: Correlations between mind-blanking and mind-wandering**.

**Experiment**	**Effect size (*r*)**	**Sample size (*n*)**	**Significance (*p*)**
Experiment 1	−0.056	23	0.80
Experiment 2a	0.172	27	0.39
Experiment 2b	−0.173	56	0.20
Experiment 2c	0.105	75	0.37
Experiment 3a	0.172	27	0.39
Sample weighted average effect size	0.029	208	

**Table 6:** **Meta-analysis: Effect of mind-blanking on behavioral outcomes (reading comprehension)**.

**Experiment**	**Effect size (*r*)**	**Sample size (*n*)**	**Significance (*p*)**
Experiment 1	−0.021	23	0.80
Experiment 2a	−0.005	26	0.98
Experiment 2b	0.056	56	0.68
Experiment 2c, part 1	0.000	38	1.00
Experiment 3a	−0.005	26	0.98
Sample weighted average effect size	0.015	169	

**Table 7:** **Meta-analysis: Effect of mind-wandering on behavioral outcomes (reading comprehension)**.

**Experiment**	**Effect size (*r*)**	**Sample size (*n*)**	**Significance (*p*)**
Experiment 1	−0.495	23	0.016
Experiment 2a	−0.400	26	0.043
Experiment 2b	−0.407	56	0.002
Experiment 2c, part 1	−0.463	38	0.003
Experiment 2c, part 2	−0.357	37	0.030
Experiment 3a	−0.400	26	0.043
Sample weighted average effect size	−0.416	206	

This mental state may often escape detection, both by outside observers and by those experiencing it. Many common behaviors can be carried out in the absence of conscious awareness, so mind-blanking may rarely be apparent to outside observers. The blank mind may also be obscured from internal self-examination; because conscious thought is the currency of introspection, any attempt to assess one's own mental states will necessarily find thoughts—and, as a result, fail to find a blank mind. This difficulty in detecting the blank mind may raise concerns related to demand effects when assessing this mental state through self-report measures (such as those used in the current research); if people are unfamiliar with the concept of mind-blanking or with the practice of assessing their own mental states, self-reports of mind-blanking may partially reflect a belief that this mental state *should* exist, rather than legitimate experiences of the blank mind.

However, data suggest that reports of mind-blanking are not simply the result of demand effects. Previous work—including experience sampling studies (Hurlburt and Heavey, 2001, 2008), and experiments focused on mind-wandering (Schooler et al., 2004)—indicates that people often spontaneously describe their minds as being “blank” in a free-response format. Results from the current experiments extend these findings. Confirmations of mind-blanking in Experiment 2a and descriptions of this mental state in Experiment 2b suggest that participants' reports of the blank mind represent experiences of a mental state distinguishable from other mental states, particularly the ostensibly similar state of mind-wandering. Moreover, the reliable lack of an effect of mind-blanking on behavioral outcomes, compared to the reliable deleterious effect of mind-wandering on these same outcomes, suggests that self-reports of mind-blanking are signals of a mental state that is not only phenomenologically distinct, but also associated with distinct underlying cognitive processes. These data suggest that self-reports of mind-blanking are, at the very least, accurate descriptions of a unique mental state; however, future research could use other measures to yield deeper insight into the nature of this mental state. For example, pairing fMRI techniques with self-report methods may allow researchers to investigate what neural processes, if any, differ between mind-blanking and other mental states typified by a decoupling of perception and attention.

Although the blank mind may be difficult to detect—at least at first—this mental state may make up much of human mental life. Evidence suggests that people's minds are removed from the present perceptual environment nearly half the time (Killingsworth and Gilbert, 2010) and that mind-blanking represents the second-most common type of attentional lapse (Watts and Sharrock, 1985). Given these findings, it may be that mind-blanking accounts for large portions human experience—whether or not people realize it.

The possibility of mind-blanking as a ubiquitous mental state is supported by research on the link between consciousness and attention. Cognitive processes generally consist of both conscious and non-conscious elements (e.g., Baars, 1997; Bargh and Morsella, 2008), and attention plays a fundamental role in determining the contents of consciousness (e.g., Crick and Koch, 1990; Posner, 1994). Attention calls into consciousness stimuli and phenomena that would otherwise remain relegated to the realm of non-conscious processing (e.g., James, 1907; Baars, 1997) and may be focused on the current perceptual environment (stimulus-dependent thought) or decoupled from the present space and time (stimulus-independent thought). The link between attention and conscious awareness seems to impose limits on the contents of consciousness (e.g., James, 1907; Baars, 1997); specifically, consciousness—defined not in terms of perceptual awareness (e.g., visual, auditory, or other sensory-based and peripheral awareness of stimuli) but in terms of verbal, representational, and/or propositional mental contents that form the center of explicit conscious awareness—may be occupied by only one flow of information at a time. It seems that whatever stimulus reaches the threshold of attention enters into conscious awareness, to the exclusion of all other environmental and intrapsychic stimuli (e.g., Crick and Koch, 1990, 2003).

Research on attention and consciousness has traditionally focused on the distinction between stimulus-dependent and stimulus-independent thought, a focus that implicitly presupposes that the attentional threshold is always met, and that people are always consciously aware of *something*. However, this assumption does not follow from any inherent qualities of the link between attention and consciousness; indeed, disregarding this unnecessary assumption allows for a more parsimonious and cognitively efficient model of attention and consciousness. People have the capacity for conscious awareness—as enabled by attention—but the possibility of experiencing mental states defined by conscious awareness does not necessarily suggest that such mental states are omnipresent. Given that non-conscious processing is almost certainly evolutionarily prior to conscious processing, non-conscious cognitive processes continue to determine much of behavior (e.g., Bargh and Chartrand, 1999), and conscious cognitive processes rely on limited energy resources (e.g., Baumeister et al., 1998), it may be that conscious awareness is the exception, rather than the rule. It may be that people's minds are blank for much of their daily lives, and attention only brings stimuli into conscious awareness when these stimuli are judged to be of potential relevance.

If the blank mind represents times when attention fails to bring any stimuli into conscious awareness, mind-blanking seems to be theoretically distinct from the seemingly similar state of mind-wandering—a period in which attention brings stimuli into conscious awareness, but these stimuli are not necessarily tied to the current perceptual environment. The current research also suggests that these mental states differ in terms of phenomenology, behavioral outcomes, and underlying cognitive processes. However, these mental states are also fundamentally related, in that they both represent a decoupling of perception and conscious awareness. Mind-wandering can be understood as a broad category of mental states, each with differing attributes (Smallwood et al., 2013), and future research could explore the ways in which mind-blanking and mind-wandering are similar, as opposed to the ways in which they are different.

Future research on the blank mind could also extend beyond identifying and measuring the blank mind to experimentally manipulating this mental state. Manipulations of mind-blanking could yield insight into what, if any, adaptive purpose the blank mind might serve. This mental state could merely fill space, acting as a placeholder when no aspects of the perceptual environment capture attention and make their way to conscious awareness, or it could serve important functions—perhaps conserving energy usually consumed by conscious attention and saving it for when it is needed (e.g., Baumeister et al., 1998), perhaps consolidating information much like a bout of micro-sleep (e.g., Born and Wilhelm, 2012), or perhaps—like laughter—acting as a mental reset mechanism when the mind is trapped in a non-sensical situation (Minsky, 1984). It may also affect behavior, either transforming individuals into mindless receptacles particularly prone to situational influences (e.g., Dijksterhuis et al., 2005) or, like a flow state, allowing people to perform intuitively without the unnecessary interruptions of conscious processing (e.g., Csikszentmihalyi, 1998).

By outlining properties of the blank mind, the present research represents a step toward a more complete view of consciousness. It suggests that the blank mind, a mental state defined by a lack of conscious awareness and enabled by a decoupling of perception from attention, is distinguishable from other mental states, including those typified by both stimulus-dependent and stimulus-independent thought. Perhaps more importantly, the present research also raises questions—not just about the blank mind itself, but also about the nature of conscious awareness more generally. A more complete understanding of the place of the blank mind within the taxonomy of mental states may allow greater insight into both human experience and human behavior.

### Conflict of interest statement

The authors declare that the research was conducted in the absence of any commercial or financial relationships that could be construed as a potential conflict of interest.

## Appendix

### Methodological details for experiment 1 and experiments 3a, 3b, and 3c

#### Experiment 1

Experiment 1 measured mind-blanking and mind-wandering during a computer-administered reading assignment using probes.

***Descriptives.*** Each participant received a total of 18 mental state probes: 6 mind-blanking probes (“Just now, was your mind blank?”), 6 mind-wandering probes (“Just now, was your mind wandering”), and 6 mind-focused probes (“Just now, was your mind focused?”); probes were answered by pressing the “y” key for “yes” or the “n” key for “no.” Probes appeared in random order, separated by a randomly generated time interval ranging from 2 to 4 min. Mean mental state counts represent the raw number of thought probes that received a “yes” response (out of six possible probes).

***Time Data.*** Probes were randomly administered by a computer program; thus, different participants received different probes at different times. In order to account for this, the 45-min reading period was divided into nine 5-min blocks. For each block, probe hit rate for each participant was computed by dividing the number of “yes” responses for each mental state by the number of probes for that state. Incidence of each mental state over time was computed as a correlation between probe hit rate and time block.

#### Experiment 3a

Experiment 3a measured mind-blanking and mind-wandering during a computer-administered reading assignment using self-catch methods.

***Descriptives.*** Participants were instructed to press the spacebar any time they noticed that they were no longer focused on the reading assignment. Participants then categorized their preceding mental state as either mind-wandering (“not only were you not really thinking about the text, you were thinking about something else altogether”) or mind-blanking (“you were not thinking about anything at all – your mind was a complete blank”). As in Experiment 2a, participants were asked to confirm their choice (“Are you sure that you were wandering, and not blanking?” or “Are you sure that you were blanking, and not wandering?”); participants answered with an equal degree of certainty for blanking (*M* = 0.86, *SD* = 0.29) and for wandering (*M* = 0.96, *SD* = 0.10) *t*_(16)_ = 1.48, *p* = 0.158. Mean mental state counts represent the raw number of self-reported incidences of each mental state.

***Time Data.*** Self-caught reports of mind-blanking were divided into twenty 1-min blocks. Incidence of each mental state over time was computed using repeated-measures ANOVAs.

#### Experiment 3b

Experiment 3b measured mind-blanking during two back-to-back computer-administered reading assignments using self-catch methods.

***Descriptives.*** Participants reported self-caught mind-blanking by clicking a button labeled “blank” below the text on the computer screen. Mean mental state counts represent the raw number of self-reported incidences of mind-blanking.

***Time Data.*** Each 6-min reading period was divided into two 3-min blocks. The influences of block and time on mind-blanking were assessed using a univariate ANOVA with trial block and time period as IVs and self-reports of mind-blanking as the DV.

#### Experiment 3c

Experiment 3c measured mind-blanking during a computer-administered reading assignment using both probes and self-catch methods.

***Descriptives.*** Each participant received a total of 10 mind-blanking probes (“Just now, was your mind blank?”); probes were answered by clicking either “yes” or “no” on a pop-up computer menu. Probes appeared in random order, separated by a randomly generated time interval ranging from 2 to 4 min. Participants reported self-caught mind-blanking by clicking on a button labeled “blank” below the text on the computer screen. Mean mental state counts for probes represent the raw number of thought probes that received a “yes” response (out of ten possible probes); mean mental state counts for self-catch methods represent the raw number of self-reported incidences of mind-blanking.

***Time Data.*** Reports of mind-blanking were dummy-coded according to assessment type (0 = self-caught, 1 = probe-caught). Each participant's responses were used to create a number string (e.g., 001010); this string reflects both the total incidence of blanking (length of string) and the method by which each incidence of blanking was assessed (value of integer). Each individual's string was then correlated with an increasing number sequence (i.e., 123456). The resulting correlations represent the degree to which the ratio of probe-caught to self-caught blanking changes over time, with positive correlations indicating that blanking is becoming relatively more probe-caught and negative correlations indicating that blanking is becoming relatively more self-caught. Individual correlations were converted to Fisher's (1915) z-scores in order to correct for the non-normal distribution of correlation coefficients. Z-scores were then analyzed using a one-sample *t*-test with a test value of 0; values significantly greater than zero indicate that mind-blanking is becoming relatively more probe-caught over time, and values significantly less than zero indicate that mind-blanking is becoming relatively more self-caught over time.
